# The Tooth-Pulp in Its Relation to Dental Operations

**Published:** 1887-11

**Authors:** William H. Trueman

**Affiliations:** Philadelphia


### THE TOOTH-PULP IN ITS RELATION TO DENTAL OPERATIONS.

READ BEFORE THE CENTRAL DENTAL ASSOCIATION OF NORTHERN NEW JERSEY, JUNE 20, 1887.

BY WILLIAM H. TRUEMAN, D. D. S., PHILADELPHIA.

I do not propose at this time to describe minutely the histology or pathology of the tooth-pulp, but rather from a clinical standpoint to consider it as a factor complicating dental operations, rendering them more difficult and painful, and seriously impairing their permanency and usefulness. I propose, while not combating generally accepted methods of practice, to give more prominence to those which an experience of more than a score of years has led me to prefer.

It is questionable if we can safely ignore the presence of a vital pulp in any dental operation; as a rule, however, where the cavity is superficial, or where it does not extend much below the enamel layer, it is seldom that the pulp is at all disturbed, yet there are exceptional cases, notably cavities upon the approximal surfaces of the anterior teeth, and those known as fissure cavities, usually found upon the masticating and buccal surfaces of the posterior teeth—cavities extending into the tooth-substance but a short distance, and which occasionally expose a sensitive tissue that tolerates the presence of the filling but little better than does the dental pulp itself. It has been suggested, and I think correctly, that this abnormally sensitive tissue, owing to some not well understood cause, has not, during the development of the tooth, been so thoroughly changed in character as has been the normal dentine, and therefore, in a measure, retains its original function. However this may be, clinically we may consider it an extension of the pulp, and in filling such cavities should use the same care and precautions usually deemed necessary in the near approach to or actual exposure of that organ, any other course seriously endangering its well being and vitality. This condition is usually confined to patients comparatively young, and is frequently associated with an organization somewhat below the normal, but is occasionally met with late in life, and in teeth of excellent structure. Many years ago, before the minute structure of the teeth and their mode of development was as well known and appreciated as it is now, Dr. J. D. White, of Philadelphia, referring to this condition, describes it as a sensitive zone extending laterally from the pulp of the anterior teeth, which he proposed to term the "pith" of the tooth. While we may not at this time accept his theory, the precautions in preparing and filling such cavities which he suggested, especially avoidance of pressure and the placing between a metallic filling and the sensitive tissue a non-conductor of heat, are as practical and important now as when he first announced them. When these are neglected, although the pain during or immediately after the operation may not be excessively severe or long continued, a degree of irritation is excited that eventually ends in devitalization of the pulp.

In its final result it thus differs from sensitive dentine; however sensitive the dentine may be, it seldom responds to simple pressure, and seldom indeed does the presence of the filling (except temporarily) prove a source of irritation. It is well, however, in all cases of excessive sensitiveness—a sensitiveness greater than normal, considering the depth and position of the cavity, especially if it seems to be confined to a portion of the cavity only—to suspect this condition and cover the sensitive portion with a cement possessing the property of not readily conducting heat, that can be applied without pressure, and that will become sufficiently rigid to protect the parts beneath when the external portion of the filling is inserted. Gutta-percha is not suitable for this purpose, as it requires pressure in its application, and does not, when so small a bulk as these cases usually permit is used, offer sufficient resistance to the pressure needful to properly insert the remaining portion of the filling. I

prefer to first flood the cavity with creosote, allowing it to remain a few minutes, dry it with bibulous paper, and use a good zinc oxychloride cement, mixed rather thin, as a protecting cap. Zinc phosphate, or the non-irritant cements prepared especially for capping exposed pulps, are less liable to cause pain, but I have found the oxy-chloride thus used comparatively painless, and have thought its therapeutic properties a decided advantage. I think it best in all cases where this condition exists, especially if the cavity is in an anterier tooth or the patient has not reached maturity, to avoid undue irritation by using a plastic filling, and keeping it thus filled for several years. A slight irritation seems to stimulate the defective tissue to further development, while but little more than this may prove destructive. This condition, in other than the central and lateral teeth, is comparatively rare. I have noticed of late that articles upon the dental pulp and its treatment have very generally ignored it. This has prompted me to give it more prominence, perhaps, than its clinical importance really demands.

In cases where the cavity approaches the pulp-chamber, the possibility or probability of inserting the filling without exciting serious irritation in the organ it contains has not seemed to me the most important question. That, in most cases, with ordinary care and the use of appliances now in general use, is readily accomplished. The real question has seemed to be this: Can we by so doing secure the best results? I do not question the relative value of a vital and a devitalized tooth. Notwithstanding the greater success now possible in treating devitalized teeth, other things being equal, the greater value of a tooth normal in its relation to the general system over one whose relation is so greatly disturbed as is a tooth from which the dental pulp has been removed, I do not consider a debatable question. This, like many surgical and dental operations, must be a compromise; a balancing of greater and lesser evils; a blending of possibilities and practicabilities; a selecting from numerous desirable and their indissolvable associated undesirable features, those which seem to promise the greater usefulness.

In treating cavities which approach closely the pulp-chamber, we have first to consider the practicability—without unduly encroaching upon the pulp—of removing sufficient of the tooth structure to secure firm retention of the filling and the thorough removal of all the tooth structure affected or weakened by caries, the presence of which would be likely to favor a recurrence of decay. In crown cavities, cavities upon the buccal surfaces and approximal cavities which do not extend toward the apex of the tooth nearer than the normal gum-line, where they are readily accessible, this is usually effected with ease. Where the cavity is deeply seated, inaccessible, upon the approximal surfaces of bicuspid or molar teeth, or extending toward the apex so far that its cervical margin is formed in cemental tissue, the difficulty is great, and the danger two-fold. We may fail, owing to the extreme sensitiveness of the tissue, to thoroughly remove all the defective tooth-substance at this point, and the inevitable recurring decay will soon render the operation of but little value; or we may, by the thorough removal of the defective tissue, without actual exposure, approach so closely the pulp that within a short time devitalization will occur as a result of the irritation excited. Bearing in mind the peculiar shape of the bicuspid teeth, and as seen in cross-section at or a little nearer the apex than the gum-line, the nearness of the pulp cavity to its outer margin, the difficulty of securing firm margins to the cavity, to so shape it that the filling may be firmly retained and yet kept at a safe distance from the pulp, or to secure these results with sufficient of a non-heat conducting lining, will be seen to be a difficult and uncertain task.

Nor is this all. Should we have, seemingly, accomplished this, knowing so well the extremely delicate organ the cavity so nearly approaches, and how very important it is to avoid any pressure upon the walls of the cavity that would cause them to impinge upon it, or in exercising the care needful to avoid displacing the cap or other non-conducting device, we are very apt not to use sufficient force to press the filling into that close contact with the walls of the cavity absolutely necessary to make a useful and permanent filling. In addition to this, we are compelled to take another risk, namely, the absolute impossibility of determining the exact pathological condition of the pulp. The absence of sensation usually indicates a devitalized pulp, or a pulp so seriously damaged pathologically that we can rarely do wrong to open into and remove the contents of the pulp-chamber. Unfortunately, the presence of sensation does not necessarily imply a vital and normal pulp; neither can we determine by the character of the sensation the condition the pulp may be in. The best we can do in determining its pathological condition in doubtful cases is a mere guess, the accuracy of which depends upon the ability of the operator to properly interpret the symptoms he observes.

Filling that portion of the cavity near the pulp with a non-conductor of heat, or with a cement which, in addition to protecting the pulp from thermal changes, shall also by its rigidity protect it from pressure during the introduction of the filling, in practice, introduces another serious element of risk. Most of the cements at present available for this purpose, used as they must be, for permanency, depend upon the protection afforded them by the filling. Although the cement properly mixed may prove, when used to form the entire filling, comparatively permanent, when mixed thin, as it must be, to serve as a lining in the cases we are considering, it is far less dense and resistant to destructive agents, especially so if they gain access to it as a result of a defect in the filling, or from recurrence of decay. The invariable result in such cases is, that as soon as from any cause it is exposed, it ceases to serve a useful purpose, and that portion of the cavity protected by it at once becomes the seat of caries under conditions favoring rapid and unobserved progress.

We have, therefore, to consider, in treating these cases, which of the two evils we will accept. First, to devitalize at once and insert a filling that we are reasonably sure will prove as permanent and protective as may be expected, considering its position and surroundings; taking the risks inseparably associated with devitalized teeth of possible and repeated attacks of pericemental irritation, that may end in alveolar abscess. (Alveolar abscess, from personal experience, unless greatly aggravated, I do not consider a serious matter. It is a condition much to be deplored, but is much to be preferred to the chronic state of pericemental irritation which often ends when it is established, and when the abscess has formed, in a great majority of cases, it causes the patient but little and unfrequent annoyance.)

Second, if we decide to fill without devitalizing the pulp, the filling may not be retained, owing to an imperfectly shaped cavity; or it may not, from causes previously stated, prove protective. In either case a devitalized pulp is the usual result, not unfrequently associated with pathological conditions that would probably otherwise have been avoided. Neither of these results may occur, and yet the usefulness of the operation may be seriously compromised by the tooth giving more or less pain when subjected to thermal changes.

I do not wish to be understood as discouraging pulp conservation or as advocating pulp devitalization, but I do unhesitatingly condemn the formulation of rigid rules regarding the treatment of cases which vary so much in their condition and surroundings—cases whose treatment depends so greatly upon the condition, physical and otherwise, of the patient, as do cavities approaching closely this delicate organ. The treatment should be left to the operator's judgment, the value of which depends upon his ability to observe, compare, reflect and record, to trace cause to effect, and to justly balance the good and the bad. The dentist who always does this, or never does that, is one whose failures are numerous; a blind and reasonless adherence to fixed rules prohibits his seeing or properly appreciating errors, and thus renders them far more numerous and disastrous than they otherwise would be; indeed, his school of experience is on vacation twelve months in the year.

In cases of actual exposure, the remarks previously made apply equally well. The question whether it is best to cap or to devitalize depends as much upon the position, extent, and surroundings of the cavity, as upon the condition of the pulp itself. The nearer the cavity of decay approaches the pulp, the more uncertain becomes the pathological condition of that organ. Where actual exposure exists, this naturally gives us the greater concern. How far its normal condition and relations may be disturbed, without impairment beyond recovery of its normal functions, is an open question. Dr. Louis Jack, of Philadelphia, at the last meeting of the Pennsylvania State Dental Society, read a paper bearing upon this question, entitled "Distinctions between the Indications of Hyperæsthesia and those of Disturbances of the Blood Vessels of the Dental Pulp," that well deserves a careful reading. Dr. Jack has so long and so earnestly advocated the conservative treatment of the dental pulp, and is so careful in collating the results of his efforts, that his conclusions, never hastily reached, are valuable. He describes, in his paper, a progressive case of pulp irritation from the near approach of decay, but first explains the peculiar anatomical arrangement of the capillaries of the dental pulp, which tend to localize inflammatory conditions and prevent their diffusion throughout the organ. He divides the observed symptoms into two sets, indicating two pathological stages, the subjective and the objective. The subjective is purely nervous, and the symptoms of a neuralgic character. During this stage the tooth responds to cold, but not to heat; pain is excited by pressure upon the contents of the cavity, but the tooth itself is not sensitive to touch, and mastication may be performed without discomfort. Conservation of the pulp may be attempted at this stage, with a fair chance of success. In the next stage the symptoms change; the tooth responds to heat, and not to cold; the circulatory system is now involved, the pain is more constant and localized, and is changed in character; the tooth is painful to touch and to pressure. When this last stage is fully developed, any attempt at treatment other than devitalization is hopeless. In a slowly progressive case there is a period when these two stages exist together; at one time the objective may be the more marked, and if, in response to treatment, recovery takes place, these will disappear and the subjective symptoms alone be observed. Just in proportion as the objective predominates, will conservative treatment become increasingly uncertain. I fully agree with these conclusions, and earnestly commend them and the paper in which they are more fully expressed to your careful consideration. I do not remember any previous attempt to isolate the nervous and inflammatory symptoms, or to make use of the marked distinctions between them in diagnosing the condition of the pulp preparatory to deciding whether to conserve or to destroy. With the special treatment of pulp exposure we have nothing to do at this time; I consider the manner of far more importance than the method.

You will have gathered, ere this, from the drift of my remarks, that much as I value a living pulp, I recognize that there are cases where devitalization is necessary, preparatory to the insertion of a reliable preservative filling; that it is necessary in some cases where, could we avoid the mechanical difficulties encountered in securing the filling and satisfy the conditions deemed necessary to arrest decay, the salvation of the pulp would be reasonably assured. There are other cases where, from the condition of the pulp, its devitalization is merely a question of time, and practically the question is narrowed down to this : Shall we apply arsenic and devitalize in a few hours, or shall we attempt to preserve its vitality with the almost positive assurance that the same result will be reached within two or three years?

For devitalization, I use arsenious acid ground up with creosote, sufficient glycerine being added to preserve its pasty consistency. A few drops of otto of rose is added to overcome the odor of creosote. This will keep indefinitely, that which I am now using being the remainder of about an ounce prepared, as nearly as I can recollect, six or eight years ago, and it will probably last to as near the next centennial as I may reasonably expect to reach. Morphia I have never appreciated in dental practice, and regard it as a worse than useless ingredient in "nerve paste." The painless devitalization of a dental pulp depends very much upon the avoidance of pressure in making the application. Morphia I consider inert, and as owing to its great difference in specific gravity from arsenic it tends to separate from it, and as it may do this so completely that the portion of nerve paste applied may consist wholly of morphia, I consider it a mischievous addition. I consider it essential to first place the paste upon a minute pellet of cotton, or in special cases, where the application must be made to the point of exposure, it is quite as essential to prick it in with a fine broach. I use as a covering a pellet of cotton, about one-fourth filling the cavity, thoroughly saturated with moderately thick sandarach varnish. This, after the application has been made, can be passed into the cavity gently and without pressure, the excess of varnish sealing it perfectly. If the paste itself is placed in position within the cavity without the minute cotton pellet to protect it, unless it is pricked in, the varnish seems to flow around it and completely protect the pulp from its action. I have used Baldocks' painless nerve paste with certain results as regards its painlessness, but very uncertain as regards its action upon the pulp. It consists of arsenic ground up with a viscid menstruum, and I am inclined to think that the menstruum protects the tissue from the action of the arsenic, much as I have found the varnish to do if the paste is not protected with a pellet of cotton before the varnish is applied. The arsenic and creosote rarely cause more than very temporary discomfort. Wax, gutta-percha, cements, etc., I consider the worst possible covering for nerve applications, and have found nothing equal to sandarach varnish and cotton.

Treatment to reduce the inflammatory condition of a pulp preparatory to a devitalization application, I regard as simply prolonging the patient's agony. The assertion so frequently made, that the inflammatory condition resists the action of the arsenic, my nearly twenty-five years of active practice has fully disproved. I have ever found that the arsenic in these cases acts as painlessly, as promptly, and as effectually as where this condition did not exist. There are cases, but few in number, however, where the application of arsenic gives violent and long continued pain, or where it fails to produce the desired effect. These quite as frequently I have found where no previous inflammatory condition has existed, as where it had. In cases where doubt exists whether a pulp is partially or completely devitalized as the result of encroaching decay of the tooth, or other causes, I see no reason why an application of arsenic should not be made, neither can I see that any harm would result from the application if the pulp were wholly dead. I have seen no injurious results from the careful use of arsenic for pulp devitalization, and can conceive of none so serious as to warrant us in attempting so barbarous an operation as the removal of a living pulp, or one whose sensibility was partially obtunded only. The idea of devitalizing and removing a portion of a pulp, leaving, perhaps, one-third of that portion occupying the pulp canal, expecting that it is vital and will so remain, I regard as "poetry," and about as practical as poetry usually is. It is true that a leg may be amputated and circulation remain perfect in the stump; but that a small portion of the tissue of a tooth-pulp in which the arteries and veins are without the usual fibrous coats shall retain its normal condition and be nourished as when the organ was intact, seems to me too improbable for serious consideration. If devitalization of the tissue be commenced, there is no certainty as to where it will end.

In the matters which I have particularly emphasized, I am aware that, while not standing alone, I differ from many whose views are often quoted or expressed in the dental journals. In some points my ideas differ widely from those usually taught. It is on this account that they are noted. They have been learned from experience, and by experience often confirmed. Many conditions and much detail that would naturally be considered under the title of the paper are omitted, from the thought that we differ but little in our treatment of them, and therefore their reiteration would be tedious and unprofitable.

